# No evidence for association between BMI and 10 candidate genes at ages 4, 7 and 10 in a large UK sample of twins

**DOI:** 10.1186/1471-2350-9-12

**Published:** 2008-02-27

**Authors:** Claire MA Haworth, Lee M Butcher, Sophia J Docherty, Jane Wardle, Robert Plomin

**Affiliations:** 1 Social, Genetic and Developmental Psychiatry Centre, Institute of Psychiatry, King's College London, UK; 2 Health Behaviour Research Centre, Department of Epidemiology and Public Health, University College London, UK

## Abstract

**Background:**

Over the last decade, associations between Body Mass Index (BMI) and a variety of candidate genes have been reported, but samples have almost all been limited to adults. The purpose of the present study was to test the developmental origins of some of these associations in a large longitudinal sample of children.

**Methods:**

For 10 single-nucleotide polymorphisms (SNPs) in candidate genes reported to be associated with BMI in adults, we examined associations with BMI in a sample of 5000 children (2500 twin pairs) with BMI data at 4, 7 and 10 years. Association analyses were performed using the Quantitative Transmission Disequilibrium Test and we corrected for multiple testing using the False Discovery Rate.

**Results:**

Despite having 80% power to detect associations that account for as little as 0.2% of the variance of BMI, none of the 10 SNPs were significantly associated with BMI at any age, although two SNPs showed trends in the expected direction.

**Conclusion:**

The lack of association for these ten previously reported associations, despite our large sample size, is typical of associations between candidate genes and complex traits. However, some of the reported SNP associations with BMI might emerge as we continue to follow the sample into adolescence and adulthood. This report highlights the importance of developmentally appropriate candidate genes.

## Background

The rise in obesity is not only seen in the adult population – obesity is also becoming more prevalent in childhood and adolescence [see e.g. [1]]. It is possible that obesity that starts in childhood results in additional health and psychological consequences [2,3]. Twin research, across the lifespan, consistently points to substantial genetic influence on individual differences in BMI and obesity [4-6]. Due to the rising levels of overweight and obesity in childhood, and the obvious long-term health problems and costs that early-onset and persistent obesity will cause, there has been an increased interest into the developmental patterns of BMI in childhood and adolescence. Very little is known about the developmental etiology of BMI and obesity in early and middle childhood. Recent research into the prevalence of obesity and overweight in an adolescent sample has demonstrated that weight is a stable phenotype by this stage of development, with little movement between groups of children classified as normal weight or overweight at age 11 and then followed up for five years [7]. Since persistent obesity is established before the age of 11, research into BMI and obesity should be targeted at younger children to assess the developmental patterns and etiology of BMI and obesity [7].

Over the last decade associations between Body Mass Index (BMI) and a variety of candidate genes have been reported [8], but samples have almost all been limited to adults. When genes are identified that are associated with complex traits such as obesity, one direction for top-down 'behavioral genomic' research [9] is to explore the developmental origins of the associations such as how early in the lifespan the associations appear. The purpose of the present study was to test some of these associations in a large longitudinal sample of children. We investigated associations with BMI in a longitudinal sample of 5000 children assessed at 4, 7 and 10 years for ten polymorphisms previously associated with obesity [8]; (see Table 1). Because genes largely contribute to stability for complex traits [10], we predicted that genes associated with BMI in adulthood would show associations with BMI in childhood, even as early as 4 years of age. The sample size provides 80% power to detect associations accounting for as little as 0.2% of the variance (p = .01; one-tailed because we only accept results in the same direction as the original reports) [11].

**Table 1:** Selected candidate SNPs

**SNP**	**Chromosome**	**Gene**	Alleles*	**Risk Allele**	**Phenotype**	**TEDS Allele Frequencies**
rs1801282	3 p25.2	PPARG	C/G (Pro → Ala)	G	Body Mass Index [33]	C = .88; G = .12
rs5082	1 q23.3	APOA2	C/T (5' near gene)	T	Waist circumference [34]	C = .39; T = .61
rs1042714	5 q32	ADRB2	C/G (Gln → Glu)	C	Body Mass Index [35]	C = .56; G = .44
rs1800206	22 q13.31	PPARA	C/G (Val → Leu)	G	Body Mass Index [36]	C = .93; G = .07
rs6659176	1 p36.11	NROB2	C/G (Ala → Gly)	C	Body Mass Index and waist circumference [37]	C = .92; G = .08
rs5443	12 p13.31	GNB3	C/T (Ser → Ser)	T	Body Mass Index [38]	C = .71; T = .29
rs7754561	6 q23.2	ENPP1	A/G (3' near gene)	G	Body Mass Index [39]	A = .77; G = .23
rs1042713	5 q32	ADRB2	A/G (Arg → Gly)	A	Body Mass Index [35]	A = .36; G = .64
rs4994	8 p12	ADRB3	C/T (Arg → Trp)	C	Body Mass Index [40]	C = .08; T = .92
rs7566605 †	2 q14.1	INSIG2	C/G (intronic)	C	Body Mass Index [21]	C = .33; G = .67

Candidate SNPs were selected based on "The human obesity gene map: the 2005 update" [8]. Nine SNPs were selected based on the following criteria: (1) Association must have been shown in a sample with at least 500 participants. (2) Measured phenotype had to be BMI/obesity or waist circumference. (3) SNP had to have a minor allele frequency greater than 0.1. Genotyping of these nine SNPs was performed by Kbiosciences, UK  using the competitive allele specific PCR (KASPar) method, on a sample of 5000 children.

* Information about the SNP function class is presented in parentheses; in the case of non-synonymous SNPs the amino acid alteration is given.

† SNP rs7566605 was genotyped in an attempt to replicate a recent report of an association [21]; genotyping for rs7566605 was performed in-house using Taqman on a subsample of 3000 children.

Full gene names:

*PPARG/PPARA*: Peroxisome Proliferator-Activated Receptors (PPARs).

*APOA2*: Apolipoprotein A-II.

*ADRB2/ADRB3*: Beta-2-Adrenergic Receptor/Beta-3-Adrenergic Receptor.

*NROB2*: Nuclear receptor subfamily 0, group B, member 2.

*GNB3*: Guanine nucleotide-binding protein, Beta-3.

*ENPP1*: Ectonucleotide pyrophosphatase/phosphodiesterase 1.

*INSIG2*: Insulin induced gene 2.

## Methods

### Sample

The sample was the Twins Early Development Study (TEDS), a study of twins born in the UK between 1994 – 1996 [12]. The TEDS sample has been shown to be reasonably representative of the UK population [12,13]. All twins and parents involved in TEDS provide informed consent for each stage of the study. Ethical approval for the Twins Early Development Study has been provided by the King's College London ethics committee, reference number: 05/Q0706/228. Response rates based on active families were 65%, 63%, and 65%, respectively at the three ages. For the purposes of the current study, we excluded from the analyses families in which at least one member of the twin pair had a specific medical syndrome or was an extreme outlier for perinatal problems such as extreme low birth weight. Data was included from monozygotic (MZ) male and female twins; dizygotic (DZ) male and female twins, and opposite-sex DZ twins. Across all genotypes the sample was 42% MZ (MZ female: 23%; MZ male: 19%), 32% DZ (DZ female: 17%; DZ male: 15%), and 26% opposite-sex DZ.

The mean ages of the twins when questionnaires were returned were 4.05 (s.d. = .16), 7.05 (s.d. = .25), and 9.93 (s.d. = .87) respectively. Zygosity was assessed through a parent questionnaire of physical similarity, which has been shown to be over 95% accurate when compared to DNA testing [14]. For cases where zygosity was unclear from this questionnaire, DNA testing was conducted.

### Measures

Height and weight data were collected by postal questionnaire from the parents when their children were 4, 7 and 10 years of age. Correlations with measured heights and weights in a sub-sample of these children at age 11 were 0.83 and 0.90. The sample included 5000 children at each age. BMI was calculated from the height and weight data (BMI = weight (kg)/(height (m)^2^)) and converted to BMI z-scores. BMI z-scores take into consideration the child's age and sex. They were based on 1990 UK growth reference curves [15] and were calculated using the program lmsGrowth (available from ). Children were categorized as normal weight, overweight or obese based on International Obesity Task Force (IOTF) criteria [16]; which are based on age-specific and sex-specific cut-off points linked to growth curves, and correspond to BMI criteria for normal weight, overweight and obesity in adults [16]. Although there are disagreements about the correct cut-off for childhood obesity [see for example [17-19]] we opted for the IOTF criteria because it was important that the cut-offs used in this study were internationally meaningful, in addition, we are not using the cut-offs for clinical purposes. Finally, the focus of our paper is not on the categorical analyses, but on the more powerful continuous analyses.

The percentages of the sample in each category (normal weight, overweight and obese) were 83%, 12% and 5% at 4 years; 88%, 9% and 3% at 7 years; and 86%, 11% and 3% at 10 years.

The mean raw BMI scores (i.e. unstandardized for age and sex) were 15.83 (sd = 1.96) at 4 years; 15.73 (sd = 1.99) at 7 years; and 17.24 (sd = 2.93) at 10 years. The raw BMIs of the 3 groups (normal weight, overweight and obese) at each age were: 4 years: 15.19 (sd = 1.29); 18.06 (sd = 0.53); and 20.95 (sd = 1.81); 7 years: 15.22 (sd = 1.29); 18.92 (sd = 0.76); and 22.53 (sd = 1.96); and 10 years: 16.36 (sd = 1.68); 21.60 (sd = 1.41); and 27.41 (sd = 5.07).

### Candidate SNP selection and genotyping

Candidate SNPs were selected based on the 2005 update of the human obesity gene map [8]. Nine SNPs were selected on the following criteria: i) the association must have been shown in a sample with at least 500 participants, ii) the measured phenotype had to be BMI, waist circumference or obesity, and iii) the SNP had to have a minor allele frequency greater than 0.1 because statistical power to detect associations is too low for alleles with lower frequencies. The SNPs that were selected are shown in Table 1, which also includes information about the genes in which they reside, the risk allele (that is, which of the two alleles is associated with higher BMI), and the phenotype of interest. Genotyping of these nine SNPs was performed by Kbiosciences, UK [20] using the competitive allele specific PCR (KASPar) method. The error rate of this method is low (< 0.5% in our analyses of blind duplicates).

In addition, SNP rs7566605 was genotyped in an attempt to replicate a recent report of an association between rs7566605 and obesity in four out of five populations [21], although it subsequently failed to replicate in several studies [22]. Genotyping for rs7566605 was performed in-house using Taqman on a subsample of 3000 children. The overall genotyping call rate was 93%, and we only called the genotypes if the TaqMan quality value exceeded 95. The allele frequencies for all 10 candidate SNPs are included in Table 1; all the frequencies in our sample are similar to those for Europeans in HapMap.

### Analyses

Results were analyzed using the QTDT total association model [23], which takes into account the twin pair structure by comparing variance between and within pairs. We did not have parental genotypes, so were unable to include IBD status in the model. The total association model was chosen after we had verified that there was no significant population stratification. In the QTDT analyses we specifically modeled a polygenic variance component, as well as a non-shared environment and twin-specific environmental variance components. Variance components were tested for significance before being included in the model. The heritability of BMI estimated by the QTDT analyses was 41% at 4 years, 60% at 7 years and 75% at 10 years.

Our sample of 5000 (2500 twin pairs) provides 80% power to detect an effect size (r^2^) of 0.2% (p = .01, one-tailed, as calculated using [11]). We corrected for multiple testing using the False Discovery Rate [24]. This multiple testing correction was applied across all ages (i.e. 30 tests) rather than at each age separately, because the measures of BMI at each age are not independent. We analyzed the entire sample of 5000 in order to maximize power to detect associations of small effect size. In addition, because the original reports compared obese and overweight cases versus controls, we also divided the sample into groups (obese, overweight, and normal) and compared genotypic frequency differences between the groups using chi-square analysis. We compared the obese group to the normal weight group and also the obese and overweight groups (combined) were compared to the normal-weight group. These analyses were done on one randomly selected member of each twin pair.

### Limitations

A specific limitation of the present study is the use of parental reports of children's heights and weights, although previous research has shown that they are reasonably reliable [25]. This was confirmed in our sample on a sub-sample at 11 years: Home measurements of height and weight, taken by trained researchers, correlated .83 and .90, respectively, with parental report. A further limitation is that we were only able to study BMI. It would have been beneficial to include other measures of adiposity, but this was not feasible in this large sample. In addition BMI was measured only at ages 4, 7 and 10 years. It is possible that we could have got different results if we had more frequent measurements of BMI during childhood, so that we could more accurately estimate when in development the association with the candidate gene emerged. However, as we found no significant associations, this limitation is unlikely to impact on the interpretation of these results.

Finally, this analysis does not consider genetic influences that impact on change during development. This could have been done using growth curve analyses, similar to Podolsky et al. [26]. However, growth curve analyses are best performed with at least four measurements, so we were unable to include such analyses in this study. All of the results should be interpreted in light of these limitations.

## Results and Discussion

We verified that none of the SNPs deviated significantly from Hardy-Weinberg Equilibrium using the exact test in Pedstats [27]. Table 2 shows the mean (and SD) of BMI by genotype for each of the 10 SNPs. Table 3 presents results from the Quantitative Transmission Disequilibrium Test (QTDT) [23] total association test. Of 30 comparisons, only one yielded a p-value less than .05. After correcting the data for multiple testing using a False Discovery Rate (FDR) of 0.05 [24], none of the associations were significant.

**Table 2:** BMI z-scores (with standard deviations in parentheses) at 4, 7 and 10 years by genotype

	**4 y N**	**4 y BMI z-score**	**7 y N**	**7 y BMI z-score**	**10 y N**	**10 y BMI z-score**
**rs1801282**						
**CC**	3670	-0.09 (1.47)	3746	-0.11 (1.15)	3696	-0.02 (1.17)
**CG**	1028	-0.07 (1.41)	1043	-0.07 (1.17)	1003	0.08 (1.15)
**GG**	65	0.02 (1.54)	58	0.38 (1.11)	63	0.38 (1.34)
**rs5082**						
**CC**	728	-0.14 (1.44)	796	-0.06 (1.15)	740	-0.03 (1.15)
**CT**	2232	-0.10 (1.45)	2289	-0.12 (1.16)	2254	0.01 (1.19)
**TT**	1787	-0.06 (1.46)	1749	-0.09 (1.14)	1762	0.01 (1.15)
**rs1042714**						
**CC**	1487	-0.10 (1.45)	1520	-0.05 (1.12)	1450	0.02 (1.16)
**CG**	2372	-0.07 (1.44)	2389	-0.14 (1.17)	2355	-0.03 (1.16)
**GG**	891	-0.13 (1.48)	937	-0.07 (1.15)	956	0.06 (1.17)
**rs1800206**						
**CC**	4160	-0.09 (1.46)	4248	-0.10 (1.15)	4192	-0.00 (1.17)
**CG**	597	-0.11 (1.40)	601	-0.14 (1.11)	584	0.05 (1.17)
**GG**	38	0.43 (1.73)	39	0.52 (1.16)	28	0.68 (1.08)
**rs6659176**						
**CC**	4033	-0.09 (1.45)	4115	-0.09 (1.16)	4056	0.02 (1.17)
**CG**	726	-0.08 (1.47)	738	-0.12 (1.10)	710	-0.10 (1.14)
**GG**	24	0.06 (1.43)	21	-0.27 (1.43)	19	0.10 (1.01)
**rs5443**						
**CC**	2388	-0.07 (1.44)	2420	-0.12 (1.13)	2406	0.01 (1.16)
**CT**	1977	-0.08 (1.47)	2016	-0.05 (1.18)	1977	0.00 (1.17)
**TT**	401	-0.19 (1.46)	423	-0.12 (1.11)	383	0.01 (1.17)
**rs7754561**						
**AA**	2766	-0.11 (1.46)	2779	-0.06 (1.18)	2713	0.01 (1.18)
**AG**	1723	-0.08 (1.46)	1786	-0.14 (1.10)	1765	-0.01 (1.14)
**GG**	238	0.04 (1.41)	252	-0.20 (1.15)	246	-0.03 (1.13)
**rs1042713**						
**AA**	670	-0.02 (1.38)	665	0.02 (1.12)	642	0.09 (1.13)
**AG**	2202	-0.10 (1.43)	2229	-0.12 (1.16)	2194	-0.03 (1.17)
**GG**	1933	-0.09 (1.49)	2001	-0.11 (1.15)	1965	0.02 (1.17)
**rs4994**						
**CC**	24	0.00 (1.42)	29	0.27 (1.39)	26	-0.29 (1.41)
**CT**	663	-0.07 (1.45)	676	-0.09 (1.10)	668	0.06 (1.20)
**TT**	3918	-0.10 (1.46)	3980	-0.10 (1.15)	3903	-0.00 (1.16)
**rs7566605**						
**GG**	999	-0.08 (1.41)	1242	-0.10 (1.14)	1401	0.02 (1.17)
**CG**	999	-0.09 (1.46)	1163	-0.11 (1.12)	1332	0.04 (1.12)
**CC**	259	-0.13 (1.41)	300	-0.16 (1.22)	346	-0.06 (1.16)

**Table 3:** Results from QTDT for BMI z-scores at 4, 7 and 10 years

**Phenotype**	**SNP**	**p-value**	**Absolute Effect Size (SD units)**
**4 year BMI**	rs1801282	0.222	0.059
	rs5082	0.246	0.038
	rs1042714	0.820	0.007
	rs1800206	0.777	0.017
	rs6659176	0.617	0.029*
	rs5443	0.316	0.035*
	rs7754561	0.624	0.018*
	rs1042713	0.440	0.024
	rs4994	0.867	0.010
	rs7566605	0.697	0.017*

**7 year BMI**	rs1801282	0.095	0.067
	rs5082	0.609	0.014*
	rs1042714	0.635	0.012
	rs1800206	0.659	0.022
	rs6659176	0.932	0.004
	rs5443	0.573	0.016
	rs7754561	0.033	0.065
	rs1042713	0.344	0.025
	rs4994	0.598	0.025
	rs7566605	0.402	0.029*

**10 year BMI**	rs1801282	0.066	0.077
	rs5082	0.704	0.011
	rs1042714	0.469	0.019*
	rs1800206	0.168	0.073
	rs6659176	0.145	0.074
	rs5443	0.809	0.007
	rs7754561	0.508	0.021
	rs1042713	0.903	0.003
	rs4994	0.683	0.021
	rs7566605	0.847	0.007*

* = effect not in the expected direction.

*Note. P-values uncorrected for multiple testing*.

The only notable genotypic differences in Table 2 involved SNPs rs1801282 (at ages 7 and 10) and rs1800206 (at ages 4, 7 and 10), which demonstrated trends in the expected direction for homozygotes for the risk allele. SNP rs1801282 showed 0.3SD greater BMI for children homozygous for the risk allele at ages 7 and 10, although not at age 4 and SNP rs1800206 showed a minimum 0.5SD greater BMI for children homozygous for the risk allele. These differences are nominally significant in comparisons between children homozygous for the risk allele and the rest of the sample (p values = 0.001 – 0.028). However, although they are in the direction expected on the basis of the previous reports, these associations could not survive a false discovery rate test. Also, because the risk alleles for these two SNPs have low frequencies (average 12% and 7%, respectively), these two genotypes are among those with the smallest sample size and thus have the largest standard errors.

We also ran the analyses separately for males and females and found no gender-specific associations, although with only half the sample we had reduced power to detect such associations. Results from these analyses can be found in Table 4. We present the p-value from the gender-specific QTDT analyses (performed in the same way as the analyses on the full sample). Before correction for multiple testing, there were two nominally significant associations for the female analyses (rs5082 at 4 years and rs6659176 at 10 years). These significant values did not survive the correction for multiple testing. There were no significant associations in the male analyses.

**Table 4:** Results from gender-specific QTDT for BMI z-scores at 4, 7 and 10 years

**Phenotype**	**SNP**	**Female p-value**	**Male p-value**
**4 year BMI**	rs1801282	0.566	0.846
	rs5082	0.006	0.807
	rs1042714	0.117	0.085
	rs1800206	0.730	0.771
	rs6659176	0.701	0.847
	rs5443	0.194	0.383
	rs7754561	0.234	0.497
	rs1042713	0.181	0.609
	rs4994	0.661	0.697
	rs7566605	0.380	0.732

**7 year BMI**	rs1801282	0.180	0.304
	rs5082	0.400	0.828
	rs1042714	0.615	0.983
	rs1800206	0.485	0.689
	rs6659176	0.479	0.505
	rs5443	0.318	0.777
	rs7754561	0.147	0.075
	rs1042713	0.578	0.464
	rs4994	0.565	0.800
	rs7566605	0.512	0.579

**10 year BMI**	rs1801282	0.235	0.336
	rs5082	0.400	0.585
	rs1042714	0.752	0.336
	rs1800206	0.229	0.214
	rs6659176	0.045	0.788
	rs5443	0.327	0.501
	rs7754561	0.170	0.914
	rs1042713	0.739	0.642
	rs4994	0.641	0.983
	rs7566605	0.217	0.111

*Note. P-values uncorrected for multiple testing*.

Because the original reports of association involved comparisons between cases and controls rather than continuous variation in BMI throughout the distribution, we also compared genotypic frequencies for obese versus normal-weight and for obese + overweight (combined) versus normal-weight, even though these are less powerful analyses. Chi-square analyses indicated no significant genotypic frequency differences after correcting for multiple testing using FDR. There were two significant p-values (< .05) in each set of 30 chi-square analyses. The p-values (uncorrected for multiple testing) ranged from .019–.932 for the analysis of obese+overweight versus controls (average p-value = .46), and from .019–1.00 for the comparison between obese and normal weight individuals (average p-value = .53). However, the two SNPs mentioned above again showed trends in the expected direction with the risk alleles showing greater genotypic frequencies in the obese and overweight groups.

These negative results reflect the larger issue that candidate gene associations have proven difficult to replicate for complex traits [28] including BMI and obesity [29]. It is likely that some of the non-replication of candidate gene associations is due to studies being underpowered to detect associations of small effect [30]. But studies with large sample sizes have also failed to replicate obesity SNPs [for example [31]], in particular SNP rs7566605 [22]. Replication in multiple samples is essential (e.g., the recent FTO gene study [32]) and may soon be a requirement by journals before publication.

## Conclusion

Although the lack of an association between these ten candidate genes and BMI in our sample is typical of association studies for complex traits, it also highlights the importance of developmental candidate genes. We cannot assume that the same genetic variants are influencing complex traits, such as BMI, throughout the developmental process. Childhood BMI is marked by change, and thus may not correspond to adult BMI, which is relatively stable over time. There is the possibility that positive associations for these candidate genes may emerge later in development as we follow these children through adolescence and into adulthood.

## Competing interests

The author(s) declare that they have no competing interests.

## Authors' contributions

CMAH performed the statistical analyses and drafted the manuscript. LMB selected the candidate SNPs, and organized the genotyping by Kbiosciences. SJD participated in the genotyping for the study. JW and RP conceived and designed the study. All authors contributed to the final critical revision of the manuscript.

## Pre-publication history

The pre-publication history for this paper can be accessed here:


